# ‘Lowering the threshold of effective deterrence’—Testing the effect of private security agents in public spaces on crime: A randomized controlled trial in a mass transit system

**DOI:** 10.1371/journal.pone.0187392

**Published:** 2017-12-06

**Authors:** Barak Ariel, Matthew Bland, Alex Sutherland

**Affiliations:** 1 Institute of Criminology, University of Cambridge, Sidgwick Avenue, Cambridge, United Kingdom; 2 Institute of Criminology, Faculty of Law, Hebrew University, Mount Scopus, Jerusalem, Israel; 3 Research Leader, Communities, Safety & Justice RAND Europe, Westbrook Centre, Cambridge, United Kingdom; University of Texas at San Antonio, UNITED STATES

## Abstract

Supplementing local police forces is a burgeoning multibillion-dollar private security industry. Millions of formal surveillance agents in public settings are tasked to act as preventative guardians, as their high visibility presence is hypothesized to create a deterrent threat to potential offenders. Yet, rigorous evidence is lacking. We randomly assigned all train stations in the South West of England that experienced crime into treatment and controls conditions over a six-month period. Treatment consisted of directed patrol by uniformed, unarmed security agents. Hand-held trackers on every agent yielded precise measurements of all patrol time in the stations. Count-based regression models, estimated marginal means and odds-ratios are used to assess the effect of these patrols on crimes reported to the police by victims, as well as new crimes detected by police officers. Outcomes are measured at both specified target locations to which security guards were instructed to attend, as well as at the entire station complexes. Analyses show that 41% more patrol visits and 29% more minutes spent by security agents at treatment compared to control stations led to a significant 16% reduction in victim-generated crimes at the entirety of the stations’ complexes, with a 49% increase in police-generated detections at the target locations. The findings illustrate the efficacy of private policing for crime prevention theory.

## Introduction

Surveillance and deterrence apparatuses take many forms. From formal social control mechanisms such as police officers and teachers to informal mechanisms such as place managers or neighbourhood watch initiatives, a ‘watching eye’ is often present in our environment. Technology is also taking part in suppressing the capacity to violate rules, with advents such as closed-circuit television CCTV and biometric measures reducing the opportunity for crime. Collectively, these devices have been contextualized as situational crime preventers, with a relatively robust model: capable guardianship can substantially reduce the likelihood of rule violations, as the increased chances of apprehension deter motivated offenders from victimizing others or their property.

As rational actors make decisions in the public domain, they are very much attuned to their surroundings, oftentimes searching for cues that would assist them in reducing the uncertainty about the outcomes of their courses of action. When an individual explores the gains and prospects of committing a non-normative act, she is particularly focused not only on the gains that lie ahead, but also on the various environmental measures that would enable a successful completion of these actions. We already know that offenders are aware of police presence, and in fact, calculate threat sanctions by the proximity of a police officer [1]. Even CCTV, which has not been found to be a strong catalyst of crime [2], can still be effective under the right circumstances; for example, stealing a car from a car park is much less lucrative when the place is heavily monitored. Similarly, school violence is less likely to take place when a teacher is physically present in the playground. Collectively, the entire model is buttressed on the premise that deterrence is possible, and prevention can be achieved through these various cues of sanction threats.

One area that has been understudied in this context is non-state policing, and specifically, the role of security guards in the prevention of crime. Despite being a multibillion-dollar industry, with millions of employees on a global level, and despite their enormous potential as surveillance agents, we still know very little about private security guards. It seems that most studies on these institutions—which have been conducted since the early 1970s—have been in the context of privatization of the state, and often with a critical eye of this process (e.g., the seminal cry on the ‘Decline of the public: The hollowing out of citizenship’ [3]). Yet how effective the implementation of private security has been in achieving their purpose has been grossly overlooked. A recent informative systematic review of the effectiveness of security guards unearthed only three before–after experiments directly aimed at the role of these agents, primarily in car parks, and none rely on randomized controlled trials [4]. This fact is quite surprising, given the enormous popularity of this industry, and the rather developed body of conceptual literature on their effect on the institution of policing, labour market, and the concept of risk society. These private guards are everywhere, and yet, their efficacy is vastly ignored.

We need to understand the cost-to-benefit ratios of these expensive devices, which ultimately we all pay for as an additional premium to the cost of living. From a theoretical perspective, however, the question of efficacy of security guards is much more substantial than ‘just’ another test of preventative measures. If it is the case that offenders are likely to rethink the idea of committing a crime simply because of the presence of these otherwise ordinary people who are wearing uniforms, a ‘walkie talkie’ and are making £8.50 per hour, then how little does it really take to deter offenders from committing crimes? If anti-social behaviour in the public domain can be reduced through private security agents, who are not officially part of the State’s social control mechanisms, then what does it say about the thresholds of effective deterrence? These theoretical questions have not yet been resolved.

To answer these questions, we set out to test the efficacy of security guards in Great Britain. We focused on one particular environment considered a public space, with a heavy footfall, that experiences a wide variety of crimes: train stations. As such, these stations are regularly under surveillance by modern CCTV, dedicated police officers and place managers, as well as by private security agents contracted for by the train operating companies. However, the effectiveness of the latter is presently unknown. Over the course of six months, we randomly assigned half of all the train stations in the south-west of England to treatment and control conditions. Eligible stations were those that official police records show experienced at least one crime per month in the year prior to the experiment. Treatment conditions were comprised of carefully monitored patrols to the station’s target locations by these security guards, as many times as possible during any given shift; whereas, control conditions were tasked to conduct themselves without deviation from their normal routine. With hand-held trackers on every security guard, we tracked the dosage of their presence: the precise number of visits and minutes that every guard patrolled at the station, in both treatment and control conditions.

Three types of outcomes were then collected: victim-generated crimes, police-generated crimes and self-reported incidents recorded by the security team managed by the train operator. Given our results, within the broader context of apparatuses that currently exist to deal with crime and disorder, we argue that private security guardianship can be explained within deterrence theory. As guards can play the role of sentinels, the manifestation of which is both a reduction in victim-generated crimes and an increase in detection, these actors are not passive environmental cues like CCTVs or extra lamp-posts. To the contrary: private security personnel in the public domain prevent crime in an active manner, much like uniformed and armed officers, and therefore should be construed as deterrent agents. As such, their contribution to crime policy has been understated and overshadowed by the critical thinking about state privatization, rather than focusing on their contribution to crime policy through their powers of prevention, detection and harm reduction.

This paper begins with a review of the available evidence on non-state policing, within the broader context of surveillance. We then progress to describe our experiment, where we provide details on our design, measures, experimental procedure, interventions and statistical procedures used to analyse the results. Finally, we discuss the findings and their implications for both theory and police practices.

## Literature review

### Theoretical underpinnings of the role of security guards in society

The widely accepted theoretical perspectives used thus far in criminology to explain the way in which non-state security officers could reduce crime, were best summarized recently [4].

*situational approaches that focus on reducing opportunity and increasing perceived risk through modification of the physical environment (Clarke*, *1995), and in perspectives that stress the importance of strengthening informal social control and community cohesion by improving the physical environment and greater investment in neighborhood conditions*

*(Taub et al., 1984; Taylor and Gottfredson, 1986)*.

Alternatives to achieve prevention in these ways were raised some time ago (e.g. theory of Crime Prevention through Environmental Design [CPTED; [5], or defensible spaces theory [6]). Underpinning these approaches is a systematic method in which the physical design of the environment can be modified, thereby reducing the opportunities for crime [7, 8, 9]). Furthermore, within this prism, situational crime prevention theory [10] is arguably the most famous subdivision of criminology [7]. This approach suggests that ecological apparatuses can be used to modify the sanction threat perceptions of offenders—namely, to reduce the attractiveness of targets. Either increasing the risk of apprehension or the effort that is needed to execute the offense can achieve this goal. These so-called socio-physical dimensions of criminal behaviour can be modified because offenders’ decisions to commit crime rely heavily on the physical attributes of the environment in which crime takes place [11].

As part and parcel of situational crime prevention approaches, surveillance has been assumed for some time now to be a driving factor. Humans can be agents of social control [12], because their presence modifies the ecology of the place. In essence, seeing and being seen—or visibility more broadly—creates a deterrent apparatus, because surveillance serves as a catalyser of crime. One underlying mechanism is utilitarian: the likelihood of apprehension increases when there are witnesses who can escalate the likelihood of the materialization of a sanction threat. Another possible mechanism, which has recently resurfaced in the specific context of body-worn cameras [13], suggests that people who are aware they are being observed are more likely to exhibit socially desirable behaviour. Whichever one of these causal, psychosocial mechanisms that explain offenders’ decision-making processes, rational actors are assumed to be deterred by these effective surveillance apparatuses. A place becomes ‘riskier’ if someone or something may recognize the offender, interrupt her or physically prevent her from victimizing the target [14].

The operative infrastructure in effective surveillance is capable guardianship [10] which is part of deterrence theory more broadly. Situational crime prevention and deterrence theory interact here, as the ‘strengthened formal surveillance’ component of ‘increase the risks’ in the techniques of situational crime prevention is very much in line with ideas of deterrence and indeed both police and security guards are given as examples of how to strengthen formal surveillance Put differently, increasing the risks through increased presence (of police or security) is but one mechanism by which situational crime prevention operates, while it is the primary mechanism by which deterrence operates [15].

As such, these ‘guards’ can be official actors (i.e. state officers), non-official actors (i.e. non-state officials), as well as the general public; as [16] commented, ‘people who can protect targets’—whoever they might be—‘are guardians’ (p. 5). They include ‘friends (as when three women decide to run together in a park to protect each other), as well as formal authorities such as private security guards and public police’ (ibid.). In fact, Marcus Felson himself did not limit the concept of guardianship to police officers [17, 18]; for him and most scholars who look at situational crime prevention [19] a guardian is one that ‘keeps an eye on the potential target of crime’ [20]. This includes

*‘anybody passing by, or anybody assigned to look after people or property […] The most important tasks for guardians are availability and monitoring. Formal or informal observers*—*for example, police officer and police CCTV on the one hand, or a large crowd and significant others, on the other, it is the idea that someone is watching and could detect untoward behaviors that deters the likely offender from committing a criminal act’*

(ibid: 6).

Thus, the literature has defended the view for some time now that, whichever the form of guardianship that is operationalized, effective and deterring guardianship that modifies the ecology of the place can create defensible spaces [6]. Yet, a small but critical distinction arises between the different theories: unlike CPTED that sets out to modify the *physical* environment to reduce the *opportunity* to commit crime [21, 12; 7; 17; 5], the men and women who act as security guards are contextualized as ‘tools of surveillance’ [22; 2], or as ‘formal authorities’ [16]. Both may reduce crime, but the latter is mainly concerned with observing and increasing the risks’ of apprehension by increasing the visibility of the sanction threat [12].

### The shifting role of security guards in late modernity

Within the latter formalization of effective guardianship enters security guards, and they represent a major shift in the security terrain across the globe. Private individuals, hired by for-profit companies that have their primary responsibly to their shareholders, primarily staff security guard positions [23]. Yet, beyond issues of privatization or differential access to security, we regard security guards as theoretically interesting because they bring to light a critical transition in the way we understand social control theory: the monopoly over policing has decentralized, with a myriad of (new) actors that play the role of state policing.

There are historical rationales behind the widespread approach of paid private security [24, 25]. For example, the public’s unfulfilled demand for ‘conventional police protection and dissatisfaction with the level of public safety in the United States led to the growth of private guard and patrol services. Although the latter have not displaced the former, in the United States the growth has been primarily in the private sector: by 1990, private police comprised three-fourths of all police’ [26].

Explaining this exponential growth of non-state policing further, police scholars [27–33], have noted in different contexts that since the late 1960s the private sector has broken the ‘tendency of criminologists and other critical scholars of a socio-legal orientation to conceptually equate the business of policing with the institution of the public police’ [30]. The reasons for this transition are based primarily on economic grounds: there seems to be cost-savings realised in the transition to a privatized social control apparatus. There has been a significant increase in the demand for private security, which has in turn, embraced a widening position in society that until very recently was considered to be exclusively within the dominion of the police [34]. Given the growing desire for defensible places, there are an increasing number of communal spaces to which police resources would normally not be allocated, and certainly not on an ongoing basis, given more urgent police priorities. Hence, paid-for, non-state policing agents, have taken over both surveillance and even investigative roles traditionally allotted to state policing agents [28]—and as such, private security agents appear to be everywhere: schools, shopping malls, airports, mass transit systems, college campuses, residential communities and parking facilities [35–40]. Thus, the expansion of private policing may have been borne out of economic necessity, or a demand from the public for additional layers of surveillance and supervision, yet ‘the bottom line’ remains the same. The presence of a privatized police force that can potentially lead to similar consequences as the state police force: less crime, disorder and fear in communal spaces.

### Key features of the effectiveness question

With these factors in mind, the question then becomes empirical: can private policing achieve these goals? Does private security reduce crime and disorder? Are they indeed cheaper than state police officers? Under which circumstances are they effective, when would they result in no meaningful outcomes or when would they backfire? Some commentators have remarked that we ‘must be careful not to exaggerate either its extent or …impact…of [the] fragmentation of policing’ [41], and we think they are correct. Despite the massive growth in private security agencies, there have not been enough rigorous impact evaluations conducted of these entities [19].

However, we can at least infer which key research questions *should* be answered, when referring to the effectiveness of security guards. We can draw lessons from parallel lines of research about the efficiency of various social control apparatuses, such as policing research. For example, the first immediate question is one of dosage: the amount of surveillance or monitoring that is likely to play a part in the assessment. Is it the case that the more of them, the more the propensity for committing crime reduces [42–46]? Offenders are deterred by observers, particularly if they are perceived as likely to materialize a sanction, so it suggests that offenders perceive committing crime as risky(ier) when there are *more* surveillance apparatuses in the offender’s and the victim’s environment.

One of the first papers to deal directly with the dosage question was [47], who found that the presence of police could maximize residual deterrence effects on crime and reduce disorder at hot spots. This observational study suggested that 10–15 minutes of proactive police patrols at these locations were sufficient to maximize the deterrence effect [48] have found through experimental methods that for 15 minutes were effective in reducing crime at Sacramento hot spots, compared to controlled conditions, although they did not observe different time intervals beyond the assigned dosage.

Recently, it has been shown that the dosage question can in fact be broken down into at least two dimensions: duration and frequency [49]. While duration implies the amount of minutes spent on patrol, the frequency question refers to the number of times the officer must visit the location to maximize the deterrent effect. Their study concluded that a greater frequency of discrete police visits may yield more crime reduction benefits than a greater duration of those visits; approximately two more 10 minute visits per day in treatment areas compared to control areas resulted in a nearly 40% reduction in crime. However, none of the available studies on the dosage question have looked at randomly allocating discrete times or visits to study conditions, so such conclusions are currently weak (however, see [50]).

How these findings translate to the cost-effectiveness question of security is currently unclear. It is possible these policing research questions and answers are immediately translatable to the world of private security. If the visible insignia of social control deter offenders [49], and if uniformed personnel carry signals of social control [50], then indeed we could assume that similar outcomes will take place when increasing the dosage of non-state police. This means that ‘more’ of them would result in ‘less’ crime and disorder. However, it may also be the case that ‘more’ of them will provide a signal to offenders that a place contains lucrative targets, which may actually increase the attractiveness of the place.

Style of delivery is also a key feature. Some commentators have suggested that ‘a guardian is any person and every person on the scene of a potential crime that may notice and intervene (*whether they intend to or not*)’ ([19] our emphases). This definition emphasizes that ‘even when the potential guardian has no intention at all to exercise guardianship, he [or she] is already acting in a passive way as a guardian by mere presence’ [19]. At the same time, not all persons are created equal, and it is logical to assume that some security guards deter more than others. Similarly, the more of a personal affinity the security guards have towards the place, the more effective they would be [51]. It seems clear that the effectiveness of these apparatuses to discourage individuals from committing crime depends on the extent to which guards feel responsible for the very place they are tasked to guard. Furthermore, would an armed patroller deter more than an unarmed officer? Is foot patrol more effective than vehicular patrol? What are the most efficient policing tactics, and for which types of places, community policing, neighbourhood policing, soft policing or plain visible deterrence? All policing styles contain an ingredient of deterrence (even an empty police car deters crime), at the very least for the duration of officers’ physical presence in the vicinity, but it may be that some styles are more effective than others for discrete sets of social control operations.

Again, the relationship between this body of knowledge and private security is unclear. We do not know, because there is no published evidence on the differential impact of different styles of tactics utilized by private security guards. In fact, there is very little known in the literature on the comparable effects of different styles of policing (however *cf*. [52], and we do not fully comprehend when policing will ‘work’, and when it will result in adverse or nil effects. Still, style of patrol is important to understand for all social control apparatuses, at the very least from an empirical perspective because different interventions have different price tags. Therefore, this question should be addressed in the future.

Ecological features seem pertinent to the effectiveness of the debate as well. The efficacy of this intervention is likely to be conditional on the characteristics of the place, in terms of the population that frequents the place, lightings [53], CCTVs [54, 55], environmental features [56] and whether or not the place is linked to night-time economy [57]. These factors can moderate the direct impact of private security, and either hinder or exacerbate the effect of non-state actors on offenders’ decisions to refrain from committing crimes.

Finally, it can be the case that effective guardianship in the form of private security would displace crime elsewhere. In her informative literature review on displacement, [58] notes two studies—[59, 60] where security guards positioned against Australian bank robberies did not cause crime to move around their corner to other targets [61]. However, these study designs did not pay sufficient attention to confounding variables and, therefore, more research is needed on this front as well.

### Evidence of effectiveness

Published rigorous evidence on security guards is alarmingly thin. The most informative source to date on the efficacy question can be found in a systematic review of the evidence [4]. The review identified only three before–after experiments directly aimed at the role of security guards in car parks [35, 37, 38] and none relies on randomized controlled trials. While two of these studies have shown measurable reductions in vehicle theft, without known geographical displacement to the surrounding areas, the third found no significant effect [37]. The non-significant outcomes were explained in the context of dosage: the intervention in inner city Rotterdam (the Netherlands) was not potent enough. More generally, as these studies did not rely on randomized trials, the potential confounding by supplementary interventions (e.g. fencing around the car parks or media campaigns about the programme) could be substantial. There may have been additional unmeasured variables that moderated the treatment effect, particularly when the studies were underpowered and focused on a handful of experimental sites—1 versus 3 in Basingstoke, UK [38]; 1 versus 3 in Vancouver, Canada [35] and 11 versus 5 in Rotterdam [37].

There are other studies that looked at the effectiveness of security guards, but their methodological rigor is perhaps more challenging. For instance, some studied investigated the effect of security guards positioned in Philadelphia banks in the United States [62]. The treatment was reported to be effective in protecting these facilities from robberies; however, this result was only based on cross-sectional analysis. On the other hand, other studied reported non-significant decreases in the number of reported street violence incidents during an intervention in a mid-size Swedish city, compared to previous years [63]. They reported no apparent changes in the number of street violence rates before and during security guard patrols in hot spots, which suggests that security guards are ineffective in reducing crime. In both studies, however, lack of appropriate control groups makes the findings somewhat suspect. These studies nevertheless teach us of the growing interest by both practitioners and scholars to test the efficacy of these actors in what have traditionally been police roles.

## The South West Trains security guards experiment

Our objective in this study is to address the void that exists in the literature in one major way: to rigorously assess the relative effectiveness of private security in the public domain. First, by experimenting *only* with the allocation of security guards to target locations, we directly test the effect of a non-state actor in the prevention of crime, as measured through official statistics. We measure the impact at specific crime locations—arguably what criminologists would refer to as ‘hot spots’ [64, 65] or risky facilities [66, 67]—as well as at entire local areas where these security guards are meant to patrol. The only difference between the two randomly assigned groups of train stations in our experiment is the amount of time spent by private police officers who carry nothing but ‘security insignia’ without any powers of arrest.

The settings in which the test is carried out—train stations—make this test particularly strong, because the observable levels of additional modes of surveillance (e.g. CCTVs, place managers) as well as formal control mechanisms (i.e. police officers) can be quantified and controlled. Train stations are also considered public spaces, because they are populated by the general public—rather than a pre-specified or privileged subgroup of individuals or social classes. We measure treatment fidelity through hand-held trackers that measured security guard presence precisely and reliably. This technology of measurement allows us to present the treatment versus control comparisons of the independent variables of the private security presence.

## Methods

### Settings and design

South West Trains invited us to conduct the experiment and provided us approval to use their employees in this study, in order to produce evidence on the effectiveness of private security in their jurisdiction. The South West jurisdiction in England and Wales includes 206 train stations, with an annual footfall of approximately 186 million (M) passengers per year (2012–2013). These stations are managed by South West Trains, which is the train operating company under license for this region of England and Wales. While the operation of these stations is given via a license to company, the stations are nevertheless ‘public spaces’, meaning that anybody can enter the premises for as long as they want and as long as they do not violate the law. That said, there are areas reserved only for paying customers, such as the platforms area, on-board the trains and the first class lounges.

The population in this region of the United Kingdom includes roughly 5.4M residents. It is primarily a rural region, with a wide coastline along the English Channel and Bristol Channel. However, South West Trains also operates within the Greater London area, which essentially means that within its jurisdictions there are, potentially, millions of residents of varying ethnic, religious and cultural backgrounds. South West Trains is a key train operating company for Surrey, Hampshire and Dorset, Berkshire, Wiltshire, Somerset, and Devon, with 1,700 trains operating daily, and approximately 220M passengers per year.

British Transport Police (BTP), who has the national mandate to police the United Kingdom’s trains and train stations, employed approximately 137 officers to patrol these South West stations. On the other hand, there are overall 30 security guards contracted by South West Trains for the purpose of maintaining order and preventing crime in these stations.

Crime is relatively low in the South West jurisdiction (crimes were recorded in 2015), with theft from person and low-harm anti-social behaviour comprising the majority of the offense categories (BTP data, 2016). However, as will be discussed below, only 41 stations experienced 80% of the total crime, with very little to only a handful of recorded crimes in the remaining 165 stations. These concentrations are similar to non-mass transit places [68, 69].

To test the effectiveness of security guards, we were granted access to BTP crime data and populated ‘target locations’. We identified that within the 41 stations with any level of crime, there are distinct target locations where crime occurs, such as platforms, station forefronts and ticket barriers. We randomly assigned 21 stations to treatment conditions and 20 to control conditions. The precise locations of the treatment hot spots and their boundaries were communicated to the South West Trains’ security team, and they were requested to send the security guards to the target locations ‘as many times as possible in any given shift’. The names of control stations were not communicated to the security teams, in order to ‘partially-blind’ them and to avoid cross-treatment contamination effects.

While we were involved in the assignment of stations into treatment and control conditions, our study does not require pre-approval of an Institutional Review Board (IRB). The security guards were not asked to perform anything that is out-of-the-ordinary, unusual, adverse, or requested to conduct activities that would place them in any sort of risk beyond the normal levels which security guards expect in this line of duty. Similarly, there was no requirement to obtain informed consent from the security guards, because the electronic tracking of their patrols, the allocation of their patrol plans, and the overall tactical and strategic procedures for security guards were identical to ‘business as usual’. Security guards are properly instructed to patrol train stations as part of their daily activities, and we did not make any significant changes—harmfully or otherwise—beyond the request to visit the target locations at increased frequencies. As with other place-based experiments intended to reduce crime and disorder, the researchers’ role was to assist in the steer of the ‘when’ and ‘how much’ of crime prevention, rather than with the ‘how’ and ‘what’ [70]. More broadly, obtaining IRB pre-approvals is unusual in this line of field research and we are not aware of similar experiments within criminology that were required to pursue such approvals. In fact, it can be argued that the study is primarily observational rather than clinical with respect to the issue of consent and IRB approvals.

### Random assignment and partial blinding

To be considered an eligible station, the station had to experience at least one crime per month over the course of 12 consecutive months. This exclusion criterion created a list of 41 eligible stations across the entire jurisdiction. We also excluded the top station—London’s Waterloo Station—as it was substantially ‘busier’ (half of the entire regional footfall), more victimized (one-third of all crimes), and heavily surveillance both by police and by private security. We conducted simple random assignment, which created a 21–20 split of stations.

Neither the police nor the security team at South West Trains were given the full list of participating stations. Communication was only made with the treatment stations and they were blinded about the location of the control stations. This blinding process decreased the chance of contamination and single-unit Stable Unit Treatment Value Assumption (SUTVA) violations [71].

### Treatment conditions

Security guards delivered the treatment: uniformed but civilian staff, with no weapons, detaining or arrest powers, and who were tasked to be highly visible. Their task was to ‘prevent crime and anti-social behaviour’, and to report any criminal activity to the security team. They were tasked to targeted specific locations (see below) that, based on official crime data, were deemed prone to crime and disorder. There were no particular times of the day that they were required to patrol the treatment stations; instead, they were asked to make as ‘many patrols as possible’ to maximize their deterrence effect through high visibility. The only quantitative direction the guards were given was that each visit to the target location should last 15 minutes, based on prior evidence about how to maximize residual deterrent effects after the security guard left the target location, as demonstrated by the ‘Koper Curve’ [68, 49, 47, 48].

The security guards were instructed to concentrate on being visible, but not to the exclusion of other tasks. As there was no relationship between the researchers and the security personnel and/or supervisors, these study-related duties were delivered to the in meetings held between the Security & Crime Prevention Manager. They were tasked to provide reassurance, to problem-solve when possible [72] and to interact with passengers in any case where they were approached. Unlike the hot spots policing experiments, the security guards were requested to actively engage with issues and with the overall public (see review of interventions in [73]. If members of the public required assistance, or if the security guards faced crime, they were still required to observe and report the event to the police. Likewise, the guards frequently and actively interacted with place managers, ticket enforcers, and sanitation staff, thus collecting basic intelligence and information on their area of responsibilities. The aim was to increase the visibility of the officers in the ‘sea of people’ that characterizes these mass transit systems and, through their high visibility gear, to deter crime, but not to neglect their other duties as surveillance agents. Still, however, these civilians were not expected to apply *any* level of force, as their legal powers are handicapped, at least in comparison to their American security staff [74, 75].

### Target locations

Researchers should use the smallest spatial unit of analysis compatible with the precision of the underlying data [76]. Similarly, practitioners have been advised for some time now to dedicate patrol resources into these smaller units, as research strongly suggests that focusing on micro-places rather than random patrols in wide areas is an effective crime control strategy [77].

We applied this approach to private policing, tested under rigorous conditions. However, the station complexes represent relatively large areas, with incredible daily footfalls. Therefore, we sought to give the security guards more focus in their patrol routines within these ‘hot stations’, which included spatial specification in the facilities and particular places that are more likely to experience crime. Our approach was therefore to locate the ‘hot stations’ out of all train stations first (n = 41), and then to focus the intervention within these hot stations more closely. In many ways, this is similar to a method of identifying ‘hot street segments’ and then focusing the intervention in particular addresses within these hot street segments that experience more crime or the more problems. We selected target locations based on three years of police spatiotemporal crime data: these crime records included a variable that indicated the precise location within the station that a crime has been reported—a particular platform, a specific shop, the concourse area or one of the bicycle sheds. We then ranked-ordered these specific ‘target locations’ and selected the ‘top target locations’ which, based on these historic data, were responsible for at least half of the reported crime at the particular station. The 50% threshold was chosen given the number of potential target locations vis-à-vis the number of security guards available to patrol (n = 30), the walking distance time between the target locations within the station and the travel time between different stations during the course of the shift (up to 1.5 hours). For example, if a particular station identified 100 crimes over the course of three years and these events occurred in fifteen unique target locations where crime, we selected *n* target locations in which half of the crime (50) was reported, and these target locations were used as fixed points to which the guards were subsequently asked to attend during the study period. The proportion of these ‘top target location’ out of all identified ‘target location’ was usually 5% (e.g., 5 top target locations out of 100 potential target locations in each station), which is similar to the upper bound of the proportion of hot street segments identified by [69] across several cities worldwide. Hence, based on this selection criteria, we were able to communicate to the security guards where crime is more likely to occur in these participating stations based on historic data, which provided the guards a more prescribed and systematic approach to patrolling the stations than they normally patrol. The use of ‘target locations was also useful in tracking the guards and the amount of patrol they were able to deliver out of the fifteen minutes assigned to each visit, as we were able to measure the delivery of the intervention more rigorously—as we explain below. From a crime prevention policy, security guards are also more likely to be seen by non-criminal elements in these crime attractors/generators [78], because while the target locations are spatially smaller than previously tested in hot spots experiments [79] they are nevertheless ‘public places’ that are more susceptible to being crime hot spots [61]. From a disorder perspective, these places are also attractors/generators of problems, as the footfall at these places is relatively high as well and, by implication, there is a greater potential for disorder incidents to occur.

To emphasize, we did not randomly assign the guards into target locations but, rather, the random assignment was at the station level. There was no practical way to allocate these so-called hot spots, because we would not be able to control for diffusion of benefits [80] given the close proximity of these target locations within the same station complex. There would undoubtedly be a spill-over effect to other target locations, to the routes that lead to these target locations and to all other areas within the station complex. Furthermore, the geographic distance between eligible stations made it impossible for the security guards to visit the target locations, on a random basis, with sufficient dosage [49, 81].

### Control conditions

The no-treatment stations were not exposed to these proactively directed security guard patrols. Nevertheless, security guards visited the controls stations frequently in a *reactive* manner—that is, ‘chasing’ incidents as they occurred. In theory, security guards should conduct proactive patrols on a regular basis. However, in practice, the busy life at train stations requires constant attention to problems, issues and addressing requests from the public, place managers and train operators. Likewise, the target locations continued to experience crime during the experimental period, and the security guards were often the first point of contact. Therefore, a more accurate definition of the intervention is an increase in ‘allocated time’ [65] where these guards focused on a ‘saturated’ presence compared to control conditions [70].

Notably, we were able to measure the time and location of all these ‘control visits’. In fact, both treatment and control stations continued to be visited by uniformed police constables both reactively (e.g. attending calls for service and responding to incidents) and proactively (e.g. stop and search, problem-solving or crackdowns). The crucial distinction between the treatment groups was that control stations were *not* exposed to a prescribed daily level of proactive patrols at the target locations by the security guards.

### Measurements

To estimate the effect of security guards on crime and disorder, we observed three types of outcomes, at varying geographic locations. First, we were granted access to police records on the number of crimes recorded by the BTP (‘999 calls’) at the designated stations (*n* = 41), and then once more at the specific target locations. These are victim-generated or witness-generated crimes, such as against-person (violence and assaults of different sort), theft from person or other property crimes. Second, we were also provided data on police-generated crimes at these two geographic levels (i.e. at the target locations and the entire station complex), which refer to detected offenses through police work, such as confiscation of narcotics or ticket frauds, following stop-and-search encounters. These reports helped us identify the extent to which the classic role of security guards to ‘observe and report’ was enhanced through their saturated presence. For both of these data sets—police-generated and victim-generated—we were able to measure the crime trends during the six months of the experiment, as well as the six months in the previous year, in order to measure the before-and-after differences at these areas.

A third outcome was the self-reported activities, per 10M passengers, generated by the security guards. These are internal records collated by the train operator’s security team, generated as the guards conducted their field patrols. Reports are captured manually in a pocketbook, and then recorded on an electronic database for each station. The data include a date/time stamp, incident type and a short description of the event. The security system used a computerized system installed shortly before the experiment commenced, so the self-reported data does not reflect the before period.

Notably, while there is some significant correlation between these self-reported incidents and the crime reports, the strength of this association is weak (*r* = .296; *p* ≤ .05). Some self-reported incidents could eventually be reported to the police, but not all. For instance, suicide attempts, ticketless riders, trespassing that does not cause delays to trains, low-level assaults against staff and minor public disorder incidents are generally not reported to the police, and can be handled by the guards at a localized level.

### Treatment fidelity

Treatment fidelity—or tracking data [82] was captured at two levels: tracking the movement of the security guards and the patrols of the police officers. For the latter, we were provided limited but valuable information: police officers were expected to conduct daily patrols of both treatment and control stations, and to complete ‘return cards’ about their achievement rates. For example, a team of officers at station X was given rosters that included up to 10 rounds of patrols per officer to the target locations throughout all hours of the day. Each officer was required to complete the return card, and to inform the police sergeant of the extent to which he or she was able to complete the roster. These return cards—thousands for the duration of the experiment—can be viewed as self-reported evaluations that officers were required to complete. We were provided access to the overall ratio of completed returns to the non-completed returns, per station—or the percentage of compliance with the roster.

Second, we calculated dosage measures in both treatment and control stations, for all 30 security guards, derived from information gleaned from the hand-held trackers. Every guard was equipped with a hand-held, Wi-Fi and GPS-enabled device that was linked to a bespoke data recording, cloud-based system, established particularly for the purpose of the experiment. Following a validation process during a pre-test phase, full deployment to all security guards was made. Practically, every time a guard arrived to a target location and then left the target location, a recorded ping was sent to the cloud-based system. This way, we were able to measure both the frequency and duration of every patrol visit to the target location [49]. Overall, we measured 17,632 patrol minutes in 4,249 total patrol visits during the six months of the experiment, with significantly more time and more visits at treatment compared to control locations (see below).

### Statistical procedures

First, we start by calculating the raw counts before and during the experiment of victim- and witness-generated crimes reported to the police and then the police-generated crimes, before and during the experiment. We captured these counts in both treatment and control conditions, and at the three possible geographic levels: at the target locations, outside these target locations, and throughout the entire station complex. For each of these six outcomes (victim-generated crimes (at target locations); victim-generated crimes (entire station complex); victim-generated crimes (all other locations in station complex); police-generated crimes (at target locations); police-generated crimes (entire station complex); police-generated crimes (all other locations in station complex)), we used generalized linear models for count data with a Poisson distribution, with the treatment condition as the parameter and the baseline values of the dependent variables as a covariate. This a similar approach taken in previous experiments with low base rates and over-dispersed data (e.g., [68, 83]. We also computed the estimated marginal means (for more on marginal means, see [84]) in order to report the mean interaction responses, adjusted for the baseline covariate (i.e., the dependent variable at pretest value) in each of the six models.

Second, we then looked at effect sizes [85] to account for the magnitude of the effects in the target locations. First, the data inputted into CMA consisted of converting these counts into odds ratios to compare treatment and control conditions across all locations, and used the corresponding 95% confidence intervals as a measure of the reliability of the estimation procedure [86]. Second, we computed the standardized mean differences (Cohen’s d; [86]) for the self-reported incidents captured by the security guards, for each incident type (trespassing, suicide attempts, public order, theft from person, assaults against staff or other passengers, and ticketless passengers). As we did not have before measures, using Cohen’s d is particularly fitting, given the distribution of our data. Note that as there is no independence between the point estimates of these outcome variations [85], we did not compute a summary effect size. We present these results as a forest plot, which helps identify the overall distribution and dispersion of the effect sizes.

Third, we conducted subgroup analyses by measuring outcome variations for particular crime types. Using the estimated marginal means, we were able to measure the differences between treatment and control conditions, within specific crime categories. As there were dozens of crime categories, we focused on the top categories in terms of prevalence—theft, violence against person, public order, ticket fraud and drugs—as these categories make up more than two-thirds of the crime in this jurisdiction.

Fourth, we measured whether there were statistically significant differences in terms of dosage, using independent samples *t*-tests. We explored these differences using raw dosage data for the frequency of patrol visits per day and the number of minutes spent by the security guards at the train stations per day, and then compared mean values. The same procedure was carried out for the proportion of completed patrols conducted by police officers, to eliminate a rival hypothesis that the results were due to the deterring effect of police presence rather than the effect of security guards. We then juxtaposed the monthly dosage data with the crime trends, during the months the experiment was conducted. Our aim here was to show whether there were visual patterns in the data that indicated changes in the independent variable were associated with changes in the dependent variable, as a function of time. We compared the trends for both patrol duration and frequency of the patrols, against police-generated incidents and victim-generated incidents.

### Statistical power

Statistical power is defined by as the probability of detecting a statistically significant difference in a comparison of two groups when such a difference truly exists [86]. As our study included 41 stations randomly assigned into treatment and control conditions, our design follows the same sample size used in some previous place-based experiments [87, 88, 48]. These evaluations—together with the other place-based initiatives covered in the [89, 79] systematic review—have collectively, and severely, detected modest effect sizes, usually around a standardized mean difference (SMD) of 0.2, which is considered low in Cohen’s terms [86]. As concluded by [48] the small sample nature of our study is unable to detect small effect sizes, ‘so we should be cautious in interpreting any non-statistically significant findings’, should such findings emerge.

## Results

### Sample characteristics

While random assignment into treatment and control conditions should in principle create two groups that are, on average, similar to one another at baseline, it is more difficult to achieve this equilibrium with smaller samples. Therefore, pretest equality needs to be tested. We used all the available and relevant indicators for this comparison (Table 1). As shown the two groups were noticeably similar to each other across the different variables at baseline; using *t*-tests to verify these differences—and by implication to test the random assignment—we show that in none of the comparisons the difference met a 0.05 statistical significance threshold. We then used bootstrapping in order to create more stringent settings for the comparison, but the nonsignificant differences remained.

**Table 1:** Baseline comparability (treatment vs. control stations).

Indicator	Treatment Stations	Control Stations	t-test statistic (p-value)	p-value in 1,000 bootstraps
N of stations	21	20		
Mean N of target locations	3.42 (2.49)^(1)^	3.45 (1.79)	.955 (.650)	0.722
Mean N of train disruptions^(2)^ (2015)	5.57 (7.65)	6.5 (8.87)	.360 (.721)	0.825
Mean assaults against staff^(3)^ (2015)	27.76 (37.56)	30.4 (32.51)	.240 (.812)	0.939
Mean N of calls for service recorded^(4)^ (2015)	208.67 (241.17)	208.75 (187.53)	.001 (.999)	0.995
Mean N of police officers^(5)^	2.62 (5.88)	3.15 (6.69)	.270 (.788)	0.731
Mean footfall (2015)^(6)^	3,977,490.67 (5,329,450.31)	5,218,917.75 (5,180,615.96)	.756 (.454)	0.710
CCTV present at station^(7)^	100%	100%		

^(1)^ standard deviation.

^(2)^ includes suicides, technical difficulties such as signalling problems, delayed trains and accidents (data provided by South West Trains).

^(3)^ data collated by South West Trains.

^(4)^ data collated by British Transport Police (BTP).

^(5)^ data provided by BTP.

^(6–7)^ data provided by Network Rail.

We note that the distribution of events at baseline values at the target locations suggests that cycle sheds (47.6%), train platforms (20.64%), shops (4.57%) car parks (3.89%) and ticket barriers (3.72%) were the most common facilities that were targeted by offenders.

Table 2 then lists our outcome variables, at the two time periods: six months in the previous year before the experiment and six months during the experiment. We report the means and SDs separately for victim-generated crimes and for police-generated detections at three geographic levels: at the target locations, in all other locations at the station complex and at the entire station level. We also present the estimated marginal means, which we will return to later below.

**Table 2:** Victim-generated and police-generated crimes counts: Means and standard deviations (pre and post random assignment), and estimated marginal mean differences.

	RA	Treatment	Control	Estimated Marginal Mean Differences (SE)
Victim-generated Crimes (at target locations)	Pre	10.38 (10.45)	9.60 (8.57)	-0.107^1^ (1.034)^^^
	Post	14.67 (12.25)	13.10 (10.95)	
Victim-generated Crimes (entire station complex)	Pre	47.67 (64.33)	25.70 (15.12)	-6.305^2^ (1.824)
	Post	51.24 (61.37)	36.70 (23.34)	
Victim-generated Crimes (all other locations in station complex)	Pre	37.29 (66.81)	16.10 (12.82)	-3.761^3^ (2.048)
	Post	36.57 (61.59)	23.60 (23.04)	
Police-generated Crimes (at target locations)	Pre	1.81 (2.34)	1.80 (3.49)	+0.955^4^ (0.359)
	Post	2.10 (3.74)	1.25 (1.65)	
Police-generated Crimes (entire station complex)	Pre	7.81 (10.41)	9.20 (7.45)	+0.244^5^ (0.748)
	Post	8.05 (9.88)	7.05 (6.92)	
Police-generated Crimes (all other locations in station complex)	Pre	6.00 (10.12)	7.40 (5.61)	-0.861^6^ (0.656)
	Post	5.95 (8.59)	5.80 (6.54)	

^1^ covariate fixed at 10.000.

^2^ covariate fixed at 28.816.

^3^ covariate fixed at 36.951.

^4^ covariate fixed at 1.805.

^5^ covariate fixed at 8.488.

^6^ covariate fixed at 6.683.

^ standard error.

Collectively, treatment and control stations (*n* = 41) experienced on average 51.6 crimes in the six-month period a year prior to the experiment (SD = 48.98), while the target locations suffered on average 14.4 crimes (SD = 12.83) during the same timeframe. The remainder of the station areas experienced 37.2 crimes (SD = 38.88)—which indicates the target locations were not responsible for the majority of crimes at the stations. Of these crimes, a mean of 36.9 crimes per station were victim-generated (SD = 48.00) and 8.49 (SD = 9.00) were police-detected crimes. Overall, theft (36.1%), ticket fraud (12.0%), public order (9.7%), assaults (8.2%) and drugs (2.9%) comprised the majority of the crime categories.

### Treatment delivery

**Fig 1:**
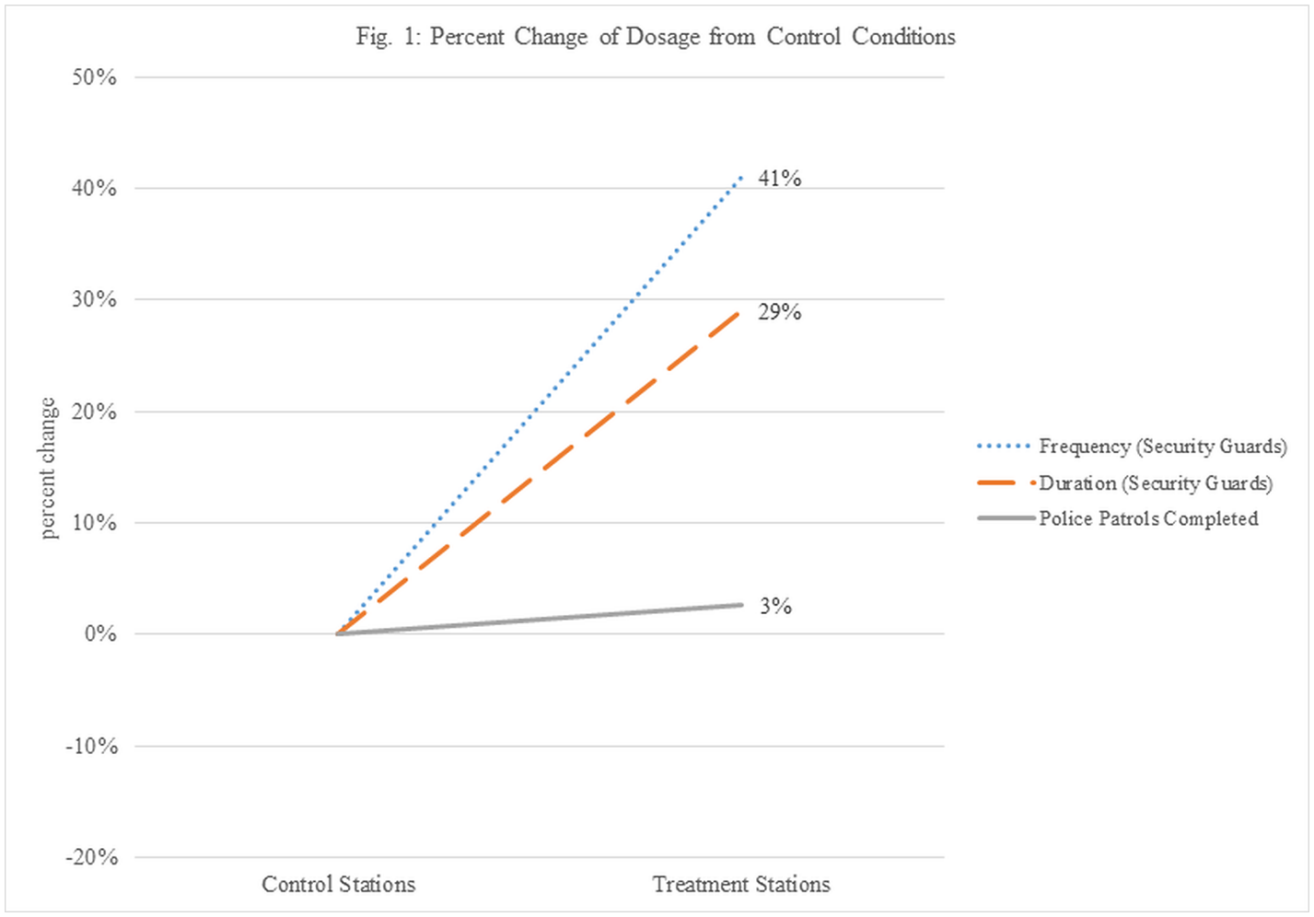
Percent change of dosage from control conditions. Percentage change between control and treatment stations in (A) the frequency of visits by security guards, (B) the duration of visits by security guards and (C) number of police patrols completed.

Based on the tracking data, it is possible to show that treatment stations received 41% more visits and 29% more patrol time than control stations. These trends in the data are presented in Fig 1 below. In absolute terms, this translates to a mean of 64.1 minutes (SD = 71) per day at treatment stations compared to a mean of 45.2 minutes (SD = 43.3) per day at control stations, and 16.3 (SD = 9.6) and 9.6 (SD = 6.2) visits, respectively. These differences are statistically significant (*t* = 3.14; *p* ≤ .001 and *t* = 8.10; *p* ≤ .0001, respectively). While the treatment stations received 11,261 minutes, the control stations received 6,370 minutes, or 2,834 versus 1,415 visits. These figures are presented in Table 3 on a monthly basis.

**Table 3:** Delivery of proactive patrols—Duration and frequency of visits, per month.

Month	Dosage (Duration in Total Minutes)			Dosage (Frequency of Visits)		
	Treatment	Control	Total	Treatment	Control	Total
March	622	178	800	115	46	161
April	3,240	1,015	4,254	762	223	985
May	2,741	1,122	3,863	529	219	748
June	1,189	575	1,764	362	182	544
July	1,032	930	1,963	363	202	565
August	981	1,046	2,027	382	294	676
September	1,456	1,505	2,961	321	249	570
Total	11,261	6,370	17,632	2,834	1,415	4,249
**Mean per day (SD)**	**64.1 (71.0)**	**45.2 (43.3)**	**t(184) = 3.14; p ≤ .001**	**16.3 (9.6)**	**9.6 (6.2)**	**t(184) = 8.10; p ≤ .0001**

We detected minimal difference in terms of proactive police patrols between treatment and control conditions (Fig 1). The average completion rate was 21.4% across the 41 participating stations, with little variation between treatment and control conditions (3% change; *p* ≥ .10). This was calculated by first subtracting the treatment value from the control value, and the dividing the change by the control, multiplied by 100 to convert into percentages. This measurement of proactive police activities in the stations provides an indication that any changes in the dependent variable can be attributed to the randomly assigned treatment condition—saturated presence of security guards—and not to the deterrent effect of uniformed police officers, or even the overall combination of both officers and security guard presence. Likewise, note that our data do not indicate police time spent in the stations while responding to crime, only the proactive patrols which the officers recorded.

### Main effects of security guards

#### Crime reports

Table 4 lists the parameter estimates for the main effects. We report the beta values, the standard errors and their associated *p*-values, for the intercept, treatment effect and outcome variable at baseline level. We report these estimates separately for victim-generated and police-generated crimes, and across the three geographic layers (at the target locations, all other locations in the station complex and the entire station). As shown, in all three estimates for victim-generated crimes, there was a crime suppression effect as a result of security guards presence (i.e. negative parameters). Nevertheless, statistical significance was reached at the entire station complex level (β = -.099, SE = .054) and areas outside the target locations (β = -.231, SE = .065), but no significant impact was observed at the target locations (β = -.009, SE = .086). On the other hand, we find an overall upward trend when it comes to police-generated crimes. This means that the presence of security guards caused an increase in detections, particularly at the target locations (β = .671, SE = .261), to some extent at the other areas in the station (β = .180, SE = .138), but no discernible effect when looking at the entire station complex (β = -.04, SE = .117).

**Table 4:** Parameter estimates.

		B	SE	p-value
Victim-generated Crimes (at target locations)	intercept	1.972	0.0778	0.000
	Treatment	-0.009	0.0857	0.917
	Pre	0.051	0.0035	0.000
Victim-generated Crimes (all other locations in station complex)	intercept	3.141	0.0452	0.000
	Treatment	**-0.231**	0.0664	0.001
	Pre	0.100	0.003	0.000
Victim-generated Crimes (entire station complex)	intercept	3.374	0.0376	0.000
	Treatment	**-0.099**	0.0536	0.066
	Pre	0.009	0.0003	0.000
Police-generated Crimes (at target locations)	intercept	0.275	0.2506	0.272
	Treatment	**0.671**	0.2614	0.010
	Pre	0.151	0.0273	0.000
Police-generated Crimes (all other locations in station complex)	intercept	1.249	0.111	0.000
	Treatment	0.180	0.1379	0.193
	Pre	0.061	0.0043	0.000
Police-generated Crimes (entire station complex)	intercept	1.388	0.0960	0.000
	Treatment	-0.038	0.1172	0.744
	Pre	0.052	0.0037	0.000

**Fig 2:**
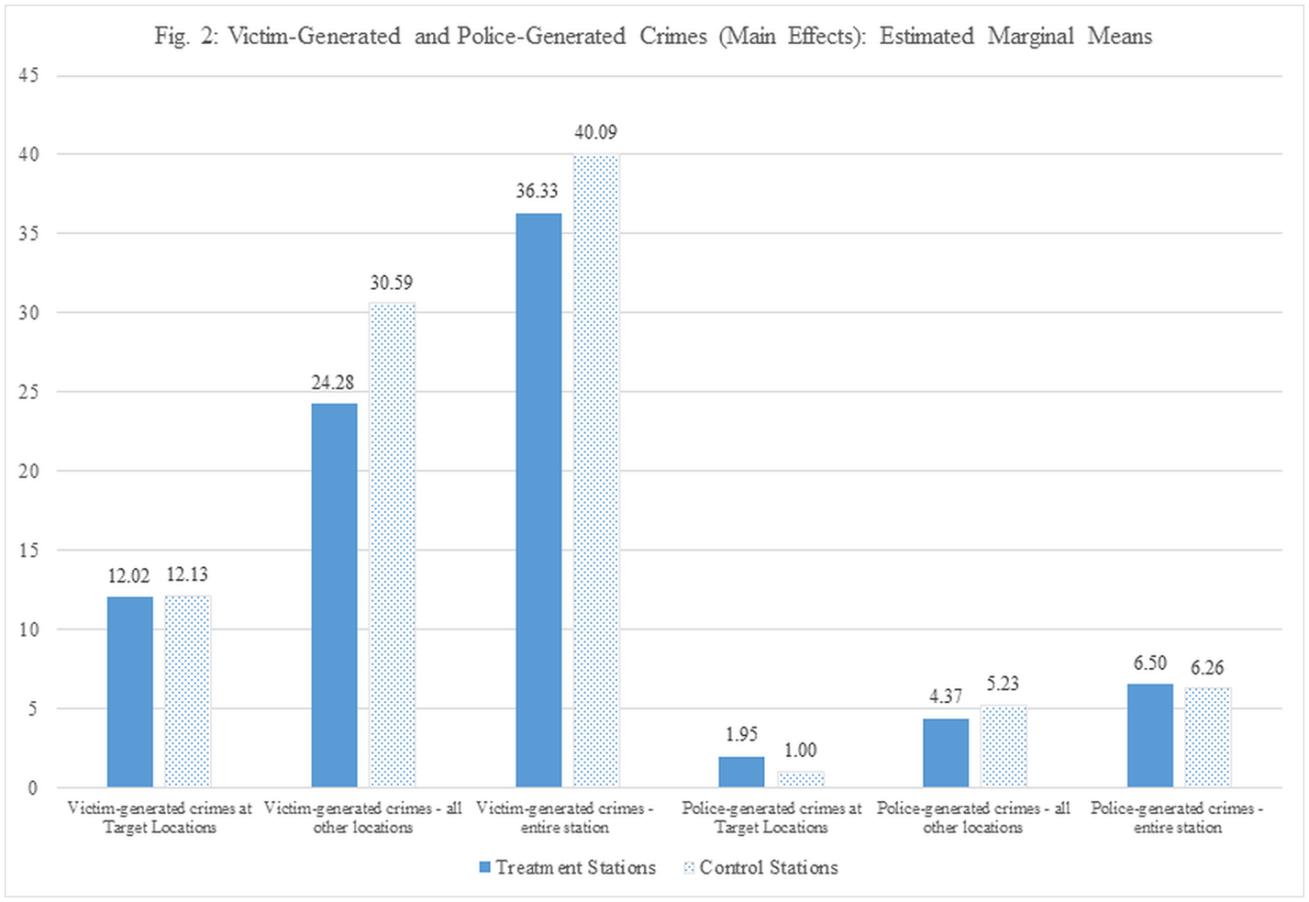
Victim-generated and police-generated crimes (main effects): Estimated marginal means. Estimated marginal means of victim and police generated of crime at differing types of locations in treatment and control stations.

Based on these parameter estimates, we then computed the estimated marginal means, which report the mean response for each factor, adjusted for all the covariate in the model. These are presented in Fig 2, and the differences in means are presented in Table 2 (right-hand column). These figures represent between 1% and 16% reductions in victim-generated crimes in the treatment group relative to the controls: the effect of the intervention increases as we move from the specific target location to the entire station complex. At the entire station complex level, the mean victim-generated crimes in the treatment group were 36.33 compared to 40.09 in control stations. In terms of police-generated crimes, the pattern is reversed: a negligible change at the entire station complex level (4%), up to a 49% increase at the target locations (means of approximately two versus one crimes).

**Fig 3:**
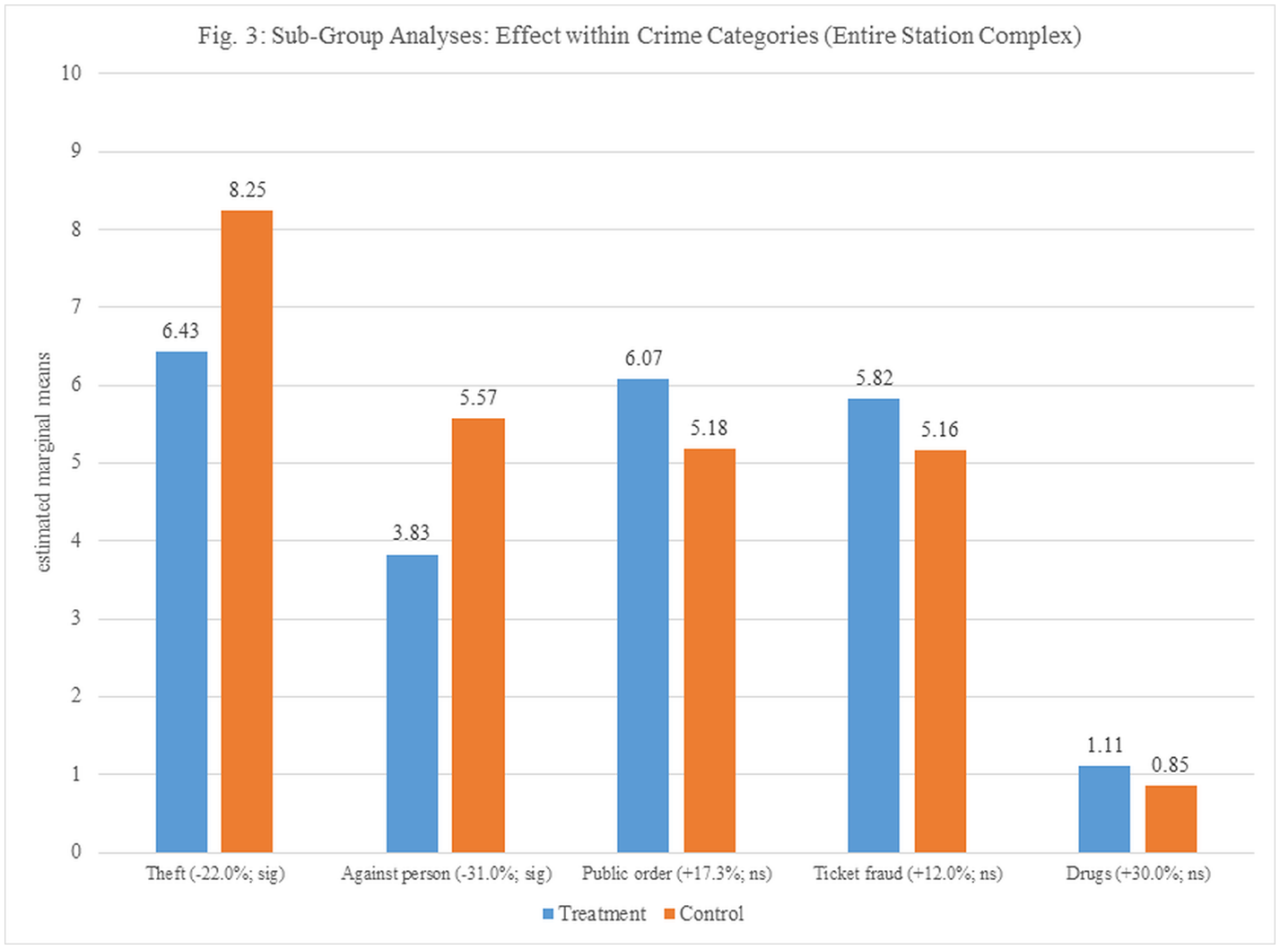
Sub-group analyses: Effect within crime categories (entire station complex). Estimated marginal means of sub categories of crime in treatment and control stations.

Outcome variations exit when observing the treatment effect on specific crime categories (Fig 3), with the most pronounced effect found for against-person offences (31.0% reduction compared to control stations), theft (22% reduction compared to control stations) and public order offenses (17.3% reduction). These are victim-generated crimes, reported directly to the police by victims or witnesses. In terms of police-generated crimes, we find increases in terms of ticket fraud and drug offenses; however, given the low base rates of these offenses (and the overall category more broadly), these differences are not statistically significant.

#### Self-reported incidents

Next, the relative effect of security guard presence was assessed based on the self-reported activities of the guards themselves, per 10M passengers. The mean scores and their corresponding SDs for treatment and control stations post-random assignment are presented in Table 5. As shown, there have been increases in reported incidents in crime categories, with varying differences, ranging from 14% for ticketless travellers up to 73% for theft. There was a mean of 1.33 per 10M passengers (SD = 2.44) incidents of thefts per treatment stations, compared to 0.36 per 10M passengers (SD = .97) incidents of theft per control stations, and 3.46 per 10M passengers (SD = 5.41) and 1.34 per 10M passengers (SD = 2.30) reported assaults against passengers in treatment and control stations, respectively. Particular attention should be drawn to the self-reported attempted suicides, with a 61% difference (13.87 versus 5.38), which we discuss below.

**Table 5:** Train operator self-reported incidents at participating stations (post random assignment only): Means and standard deviations per 10M passengers.

Incident Type	Treatment (n = 21)	Control (n = 20)
Public Order	16.54 (23.21)	6.86 (7.62)
Theft	1.33 (2.44)	0.36 (0.97)
Trespassing	12.94 (12.53)	6.09 (7.7)
Attempted Suicide	13.87 (18.9)	5.38 (9.28)
Ticketless	1.83 (5.03)	1.58 (3.15)
Assaults against passengers	3.46 (5.41)	1.34 (2.30)
Assaults against staff	4.47 (5.62)	3.80 (4.68)

**Fig 4:**
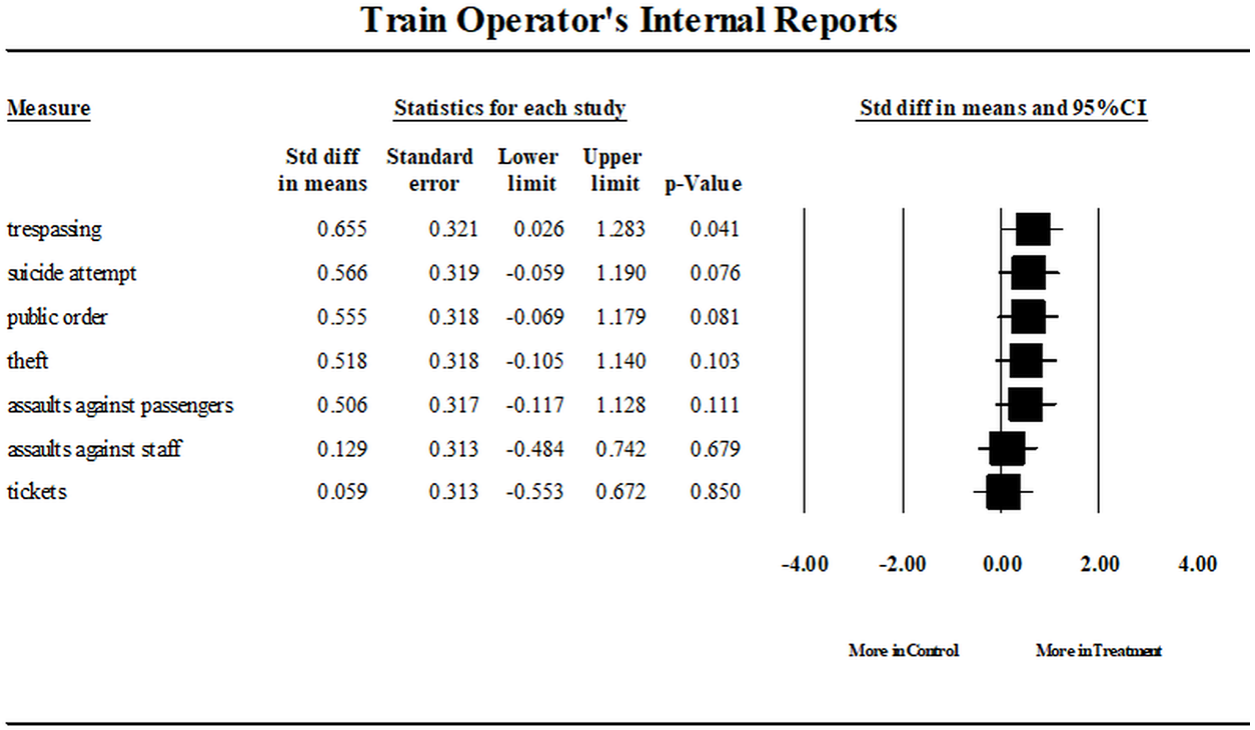
Train operator’s internal reports. Standard difference in means in categories of incidents recorded by train operators, compared between treatment and control stations.

To measure the magnitude of these between-group differences, effect sizes were computed. These analyses are presented in Fig 4. Attention should be drawn to the forest plots (right-hand side). Each point estimate represents the magnitude of the difference between the groups in terms of particular incident categories. Anything to the right of the null line (0.00) implies that the difference favours the treatment group and to the left of the line indicates that the control group performed better. The further away the point estimate is from the null line, the larger the magnitude of the difference. Once the mutual variability is taken into account, medium effect sizes (Cohen 1988) were detected for trespassing [d = .66 (95% CI .026, 1.28)], suicide attempts [d = .57 (95% CI -.06, 1.19)] and public order offenses [d = .56 (95% CI -.07, 1.18)]. Small effect sizes were detected for theft [d = .32 (95% CI -.11, 1.14)] and assaults against passengers [d = -.31 (95% CI -.12, 1.13)]. Finally, we have counted no significant increases in reported incident of assault or abuse against the security guards [d = .13 (95% CI -.48, .74)].

### Juxtaposition of dosage data and crime trends over time

**Fig 5:**
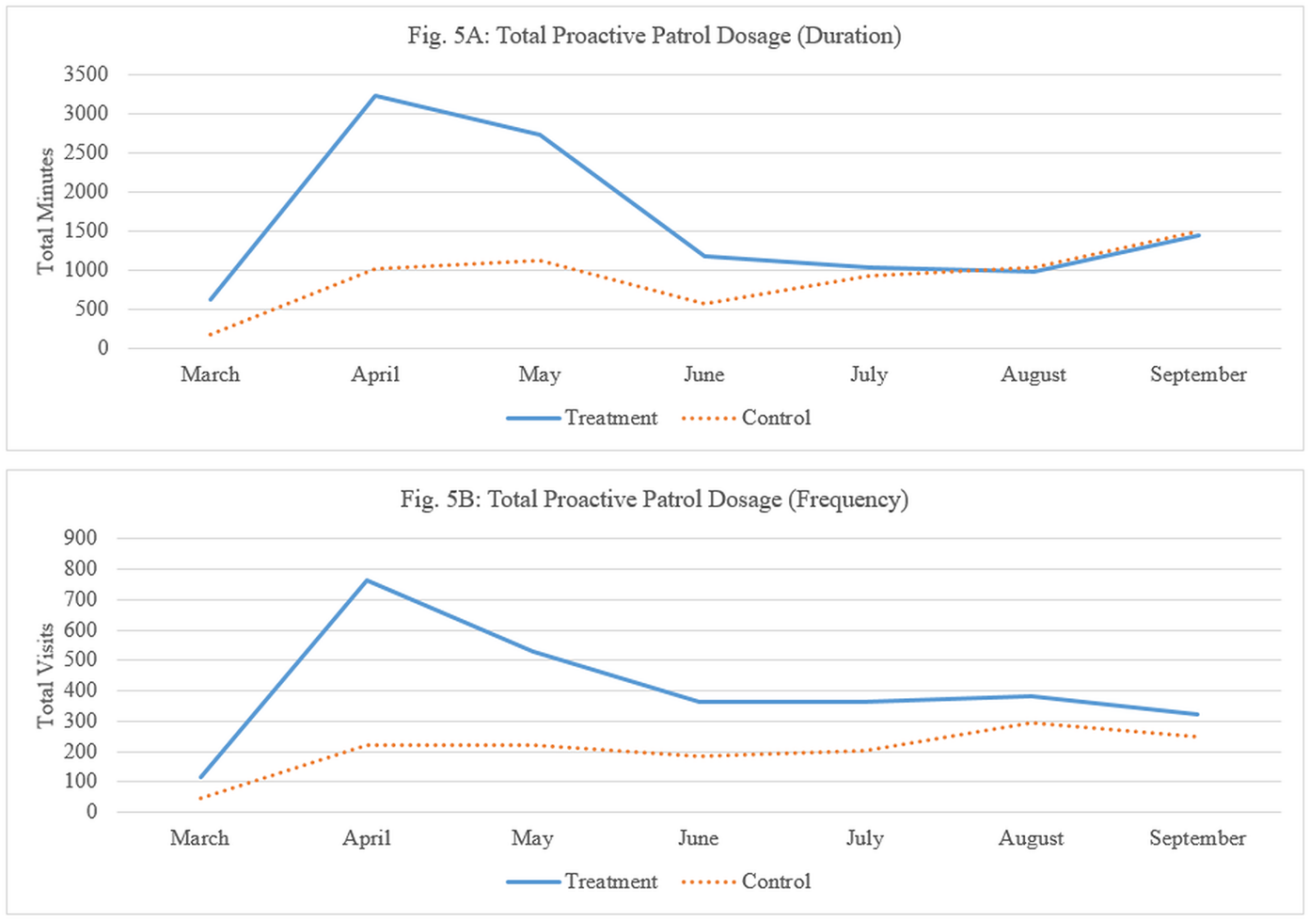
(A): Total proactive patrol dosage (duration), (B): Total proactive patrol dosage (frequency). (A) Total number of minutes of proactive patrol, by month, in treatment and control stations. (B) Total frequency of proactive patrol, by month, in treatment and control stations.

**Fig 6:**
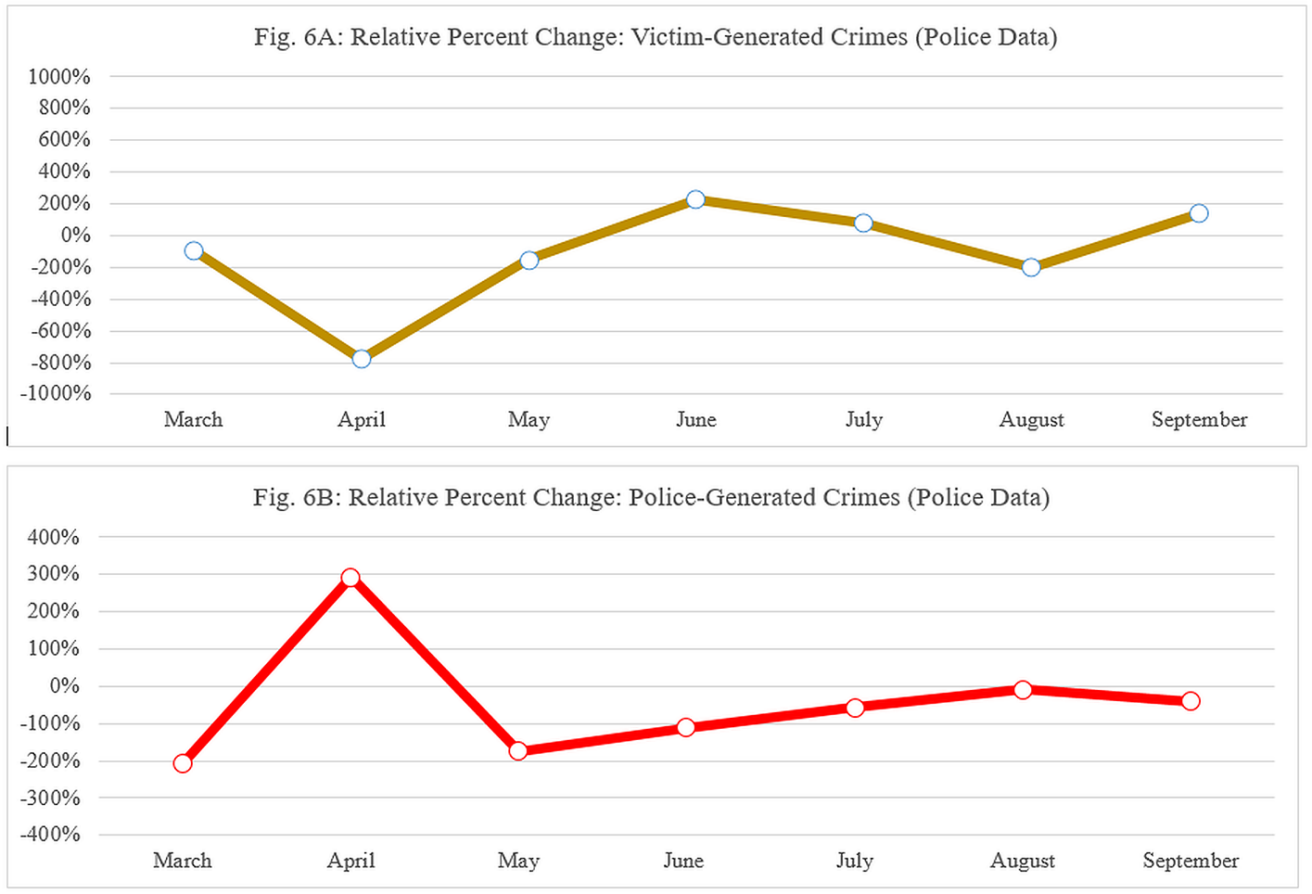
(A): Relative percent change: Victim-generated crimes (police data), (B): Relative percent change: Police-generated crimes (police data). (A) Percentage change in victim generated crimes recorded by the police at treatment and control stations. (B) Percentage change in police generated crimes recorded by the police at treatment and control stations.

Our final set of analysis was conducted to provide further support for the causal mechanism: we contrasted the dosage data—both the frequency of patrols and the duration of patrols—with the victim-generated and then the police-generated crime figures. These are presented in Figs 5A, 5B, 6A and 6B. The data are presented as a per cent change between treatment and control stations, which means that positive per cent changes indicate an increase in the treatment stations (either dosage or crime), while negative per cent changes refer to decrease in either dosage or crime in the treatment compared to control stations. The data are presented on a monthly basis (March–September).

Fig 5A and 5B suggest that while there were some negligible differences over time in terms of total proactive patrol dosage in control stations, treatment stations were exposed to a dramatic increase in dosage during the three initial months of the experiment. However, as time moved on, the relative increase in dosage diminished, until there was virtually no difference between treatment and control conditions towards the end tail of the trial. Similar patterns emerged in hot spots experiment [70], which we discuss below. Nevertheless, there is a clear indication that security guards have made the most dramatic increase in both frequency and duration during the months of April and May 2016, compared to control stations.

Fig 6A and 6B show opposite trends during the beginning of the trial, and then plateauing around the nil effect (horizontal 0% change line). On the one hand, we see a reduction in victim-generated crimes, with a particular dip during April and May (up to eight times fewer), as compared to control conditions. On the other hand, there is an increase in detected (but not solved yet) crimes during the same months (up to three times more), as compared to control conditions.

As shown, juxtaposing the first set of graphs with the second set of graphs suggests a strong match between the patterns of change from month to month: as dosage increases (both frequency and duration), there is a reduction in victim-generated crime (Fig 6A), but an increase in the police-generated crime. Both time and patrol visits are positively associated with crime detections and negatively associated with new victim-generated crimes.

## Discussion

Since the 1970s there has been a substantial increase in the utilization of non-state social control agents. From surveillance cameras and alarms systems to ‘smart’ lightings and security guards, this multibillion dollar, global industry is expanding. Security guards are already part of popular culture (for example, guards appear regularly on Hollywood blockbusters such as *Night at The Museum* and television shows like *The Simpsons* and its Springfield Mall security guard). They are funded by local governments; they take an active role in protecting our children; ensuring national security; and safeguarding our monetary institutions. Despite its growth, scholars in varying disciplines are split in their views about the appropriateness of what has become a social institution. While some see great societal benefits in having them around, others see security guards as strong symbols of the privatization of the modern state, where the privileged can seclude the underclasses and keep the masses away from gated communities. Others merely see them as another tool in widening toolbox of social control apparatuses, as a method of enhancing surveillance within an ever-growing risk society, or as an outlet to expunge unwanted or unattained police functions.

Whether or not these scholars like it, the presence of for-profit security guards among us will continue to grow. As such, we believe there are two sets of scholarships that should be considered appropriate (and rather overdue): first, there ought to be a line of empirical inquiries about the outcome of deploying security guards in the public domain. For example, are security guards efficient in reducing crime and disorder? What is the working relationship between state-police and non-state-police? Are guards cost-effective and do they therefore justify their presence? Despite the existence of an informative systematic review [4], we were left with weak responses to these empirical dimensions. The available evidence has not been sufficiently rigorous.

The second, but more substantive, set of questions should explore the wider theoretical context in which we ought to understand the (potential) contribution of these social control agents. The contribution of our work goes beyond practice and can serve those who study how offenders interpret risk perceptions, thresholds of effective deterrence mechanisms, and choice models more broadly. The role of patrolling non-state agents in the public domain has largely been overlooked in the conceptualization of deterrence theory.

### Contribution to social control theory and practice

To summarize the results of our study, this field trial involved randomly assigning entire train stations in South West of England, with an impressive footfall (188M passengers per year) into treatment and control conditions. Over six months, 30 private security guards were tasked to proactively patrol target locations, which were selected based on official crime records. We looked at several outcome measures and at different geographic levels within the station complexes. The statistical analyses suggested a significant 16% reduction in victim-generated crimes at the entire station complexes, with a 49% increase in police-generated detections at the target locations (30% increase in drug arrests and 12% ticket fraud offences).

What do these findings mean for social control theorists? The most direct question is the extent to which private security guards can take on at least some of the classic roles of ordinary police officers. On the one hand, there is a long list of legal powers that only police officers hold, which private security guards are unable to exercise: arrest, and detention powers, the capacity to (deadly) use of force, power of entry into private facilities, and requiring certain people to give them information, among others. There is a level of training that a common constable goes through, both at entry level as well as continuing professional training. In many police departments, there is already a requirement for a bachelor’s degree for new recruits. For the most part, at least for the type of private guardianship we studied in England, none of these features characterizes security guards.

On the other hand, if the results of this study are credible, then our findings suggest that, *prima facie*, security guards are capable of materializing some of the manifestations of state policing. As measured by the official records collated by the police—both victim-generated and police-generated—security guards prevent a wide range of criminal activities from taking place; they lead to the detection of notifiable offenses; and they can manage initial crime scenes.

Furthermore, we are able to identify the relative effectiveness of security guards. As we used an experimental design with a strong system of measurement of dosage, the outcomes provide a practical guide to social control theorists as well as practitioners—perhaps more than any other pervious study of place-based interventions (including the hot spots experiments). We tracked the movement of these guards, as well as the police that patrol these stations, using GPS and Wi-Fi devices, and have shown that while no discernible differences were detected for police constables, the security guards made 41% more patrol visits and spent 29% more minutes at treatment compared to control stations. There were no discernible changes in police constables’ dosage at the treatment stations. We also found a correlation between dosage and outcomes: fluctuations in patrol delivery mirrored crime trends at the participating stations—which strengthen our conclusion that the presence of security guards had led to a reduction in crime and disorder.

These dosage delivery measures can be translated into cost-effectiveness measures, albeit crudely. The treatment stations received 82 *more* hours and 1,419 *more* visits, compared to control stations, during the six months of the experiment. Spread over the 30 security guards seconded to this project, the cost of the additional patrols that focus on the target locations means 45 minutes, per officer, per month. As the average security guard at train stations in the United Kingdom is about $10 per hour (https://www.indeed.co.uk/Station-Security-jobs), the total cost of this project for South West Trains is $820. Put differently, with less than one hour per month per security guard, and with a total cost of $1,640 per year, private security can reduce against person crimes by 31% and theft-from-person by 22%. Hence, even though this is an already low-crime environment, a low-cost intervention such as private security can reduce many crimes.

Of course, ‘police work’ is complicated, multifaceted and riskier than that of a security guard. The level of public demand from a cop is entirely different from what we would demand from a private agent working for minimal wages and potentially limited affinity to the job. Being a police officer is a career choice while a security guard job is usually a temporary employment. We do not argue that one can—or should—replace the other. However, we do argue that security guards are efficient actors who can take an active part in the diversification of the modern criminal justice system portfolio. They are able to reduce crime and disorder in the public domain with relatively low costs. In doing so, they are able to ‘free up’ some of the limited time and resources that constables have, who in turn can do other, more complicated tasks. Non-state actors can successfully deliver ‘visible guardianship’, so it seems. We have not tested the *relative* success of these patrols vis-à-vis police constables, nor looked closely at how constables and guards work together; however, without changing the dosage of policing in treatment or control conditions, we do show that saturated presence of guards causes crime control benefits.

‘Police may often create only marginal impacts from incremental policy changes’, conclude [49] but the evidence tends to suggest that the police is not alone in its capacity to deter offenders. It seems that the quantity of deterrence is not just about visible *policing*, but also—and more broadly—about how much ‘symbolic quantification’ of power is generated by power-holders [90, 91] The risk of apprehension by *any* sentinel is associated with a degree to which power-holders are perceived as capable agents of rules and norms [92, 93]. Therefore, provided the capable guardian is not considered ‘toothless’ [94] by the offender, the presence of a power-holder sends out a signal: beware! In the context of deterrence theory, this implies that the perceived costs of the offender links between the symbol and the potential victim negates the appeal to commit crime [95, 96]

To emphasise, it would be a mistake to look at each organ—privatized police and state police—as working in siloes. While private police have indeed expanded and will continue to grow in the foreseeable future (G4S, 2016), the two share responsibilities, sometimes even for the same. For instance, scholars have compartmentalized public policing into ‘high’ and ‘low’ policing [97]. The former relates to policing public protests, criminal networks, terrorism and major public disorders; the latter refers to ‘ordinary’ street crimes, disorder and general help to the public. This distinction is important, because while high policing is more centralized and ‘hard-nosed’ [30] low policing is more service-oriented, ‘softer’ [49] and community-oriented. One could see that security guards and other forms of private surveillance apparatuses would be fitting within low policing strategies. As such, we are likely to see crossovers between privatized policing and low policing, with high policing reserved to public policing. Such distinction also sits well with the self-identify of many police officers who view themselves as ‘hunters’ [68, 15]

Thus, the same communal space, with clear demarcation in some types of policing, but converging roles in others, where private policing embraced public policing, and vice versa, working together to manage risk in society [98–100]. One must also take into account the age austerity that the British government is currently suffering and expecting to endure in the foreseeable future. If *some* of the cost of public space security can be reduced, then private guards may very well provide a valuable service that has been somewhat overlooked. It seems that much of the debate on the cost-to-benefit ratios of security guards was handicapped, given the lack of tangible and causal evidence of their contribution. If security guards can supplement basic crime control functions, with less cost than the operational cost of state agents, policymakers should consider new policing models that formally incorporate for-pay guards in the overall crime suppression strategy and tactics. Future research should take a closer look at this question, considering the organizational implications, financial dimensions and inter-agency collaborative roll out.

### Contextualizing the effect: Deterrence

Our second and possibly more critical contribution to criminology is theoretical: As security guards take an *active* role as deterrence sentinels [15] and as they take direct and immediate action to prevent and pursue offenders, they are no longer static and reactive environmental agents of change; they are dynamically engaged in law enforcement. They are actively engaged in visibility and providing reassurance. By being present, some crime reports increase at target stations, as people might be more likely to report crime. However, and more profoundly, the capacity of security guards is to prevent crime and disorder, which is precisely what proponents of deterrence theory envisage when they operationalize the psychosocial and environmental mechanisms of prevention. Hence, security guards are agents of deterrence, in similar ways as police officers—at least in the context of prevention and detection of some street crimes.

For example, we can draw clear parallels between ‘police deterrence’ and ‘security guards deterrence’, because our findings elegantly communicate with the latest evidence on the role of police officers in deterring offenders [70, 15, 101, 65]. By now there is ample evidence that the perceived certainty of punishment is causally associated with less crime [102, 103, 104, 105, 106, 107, 108, 109]. It appears that increasing the likelihood of apprehension reduces the odds of offenses being committed. This is the ‘certainty effect’ [15]. Within ‘police deterrence’ research, overt and *uniformed* power-holders can make arrests for criminal transgressions they detect, and they, in turn, cause rational actors to become substantially less likely to commit crime. It is the manifestation of power that power-holders exert that causes a deterrence threat, rather than the *immediate* potential for arrest [49]. The reason why deterrence cues work on offenders is what as [110] interpret as subjective probabilities that potential offenders assigns to apprehension. For this reason, even various forms of ‘soft’ policing [111], including unarmed community police officers, can deter crime, let alone more aggressive styles of paramilitary policing tactics. Deterrence theory also elegantly explains place-based policing [70]. This scholastic enterprise has shown that focusing on specific target locations is particularly effective in reducing crime and disorder, through focused efforts. As offenders become aware that police officers are presently available to cause their arrest, the likelihood of crime and disorder is substantially reduced [79]. The operative mechanism is the offenders’ heuristic approach to committing crime [68]: the unpredictable movement of police officers in space and time creates defensible places, because the potential offender cannot calculate the probability of apprehension. Thus, a hot spot no longer contains attractive targets, as the police officer’s presence or *potential* presence, deters.

Enters the security guard: based on the evidence, s/he can exert deterrence cues, because by definition the security guard increases the likelihood of apprehension. This includes not only a preventative measure—that is, reducing the attractiveness of suitable targets as ‘someone’ is there to report on the offender’s behaviour—but a fundamental increased risk of capture. All forms of security guards are deployed to protect property and life, especially by preventing unlawful or undesired entry from individuals that are not entitled to use the defended space. Within this context, we find an elaborate and rather mature literature in the context of the privatization of the state [26]. Broadly speaking, this body of research is critical of the role of non-state police, suggesting that the privatization of urban space has “raised concerns, as a process that systematically sorts the privileged or compliant from the undesirable or disobedient […], while subjecting those who patronise these forms of ‘mass private property’ […]to forms of surveillance and social engineering so pervasive that conformity to their rule systems is induced unthinkingly” [34]. The view of these security guards is such that creates “ontological security” apparatus ([112], p.2) which in turn compartmentalizes the world into “bubbles of governance” [113]. These privatized surveillance apparatuses are used to concretize the economic inequality in society, by limiting access of the underprivileged to spaces populated by majority parties in society. As important as this line of research may be, it is beyond the scope of the present study. Yet this goes beyond a subtle surveillance role: they do so dynamically, proactively and engagingly.

Much like the police officer, a security guard makes hot spots less susceptible to crime and disorder on two levels. First, as offenders become conscious of their physical surroundings, a visible security guard conveys a deterrent threat of immediate or close-to-immediate apprehension. As security guards often do not work in silos—at least not in mass transit systems in the United Kingdom—a police officer is usually around to detain or arrest a suspect. This is particularly the case with the target locations as constables frequently patrolled on a daily basis the hot spots we attempted to cool down. Therefore, committing an offence in the physical presence of a security guard is unlikely to be a preferred modus operando for a rational actor.

The second, and arguably more interesting, manifestation of private security deterrence is the effect that takes place when guards can *potentially* cause the apprehension of offenders. As we have found in this experiment—and, again, not entirely different from police-led, place-based experiments—there seems to be a residual deterrence effect [114], at least on two spatial levels. First, there was no increase in crime at the target locations, as compared to control conditions. This deviates from previous studies on the crime reduction initiative in bus stops across London [68]. In explaining the *increase* rather than decrease in crime at bus stops following *regimented* police visits, their argument suggested offenders can monitor such micro-spatial crime hot spots, and therefore offenders can predict the whereabouts of the police. Therefore, hot spots policing can backfire when offenders can calculate the true probabilities of apprehension. Yet this is not the case in our experiment. As security guards were not instructed to visit the target locations at any given time during the day, the unpredictability increased the certainty effect (or from the offenders’ perspective, reduced the certainty of a successful crime event). Therefore, crime and disorder did not go up as a result of random patrol visits to target locations, during the moments when the security guard was physically not present.

Second, we have found non-marginal effects in the areas leading to the target locations within the treatment station complexes, compared to control conditions. The most straightforward interpretation of this is what [80] refer to as the diffusion of benefits of crime control initiatives. Much like [58], [61] and the informative meta-analysis on diffusion of benefits [67], we again do not have informative evidence to suggest that crime moves around the corner, even at the level of train stations. Instead, we see the radiation of deterrence [115] The psycho-environmental mechanism seems clear: as the offender does not have access to the roster and patrol strategy of the security guard, he cannot know where the ‘target location’ begins and where it ends, nor does he know the dosage externalities the security guard is about to exert. Therefore, from the offender’s perspective, the entire station complex is now risky, and the measurable outcome of these decisions is a reduction in crime and disorder.

One final observation in this regard is that there does not seem to be confusion about who is a police officer and who might be a security guard. While we did not conduct surveys with the offenders or passengers, there is evidence to suggest that the public can distinguish between the various actors. Even though ‘they’ all conduct foot patrols, and often wear many tools, formal insignia and aim to appear formal, the British public can clearly differentiate between the different actors [116]. Participants in surveys can tell that they provide weaker control signals visually and in terms of their roles. Yet despite this awareness, the security guards are *still* able to prevent crime and disorder. Offenders are deterred by symbols of legitimate power-holding, even when they are considered a ‘lesser’ authority [50].

### Additional limitations and future research avenues

We hold the view that mixed methods and direct evidence on decision-making processes are needed to provide more robust responses to some of the questions we raised earlier. We made assumptions about the ways in which offenders perceive risk, and we discuss their decision-making processes at an abstract level. While we believe the findings are robust, much like experiments in criminology more broadly, we are only able to report on objective crime variations, as collated by the police and the security guard themselves, rather than evidence on the psychosocial mechanisms that play part here. We have also did not incorporate additional variables that could provide insight of additional mechanism at play, such as the interaction effect between the police and the security guards (i.e., ‘force multiplier’; see [115]), crime patterns outside the station boundaries, as well as the ecological factors that could have mediated the strength of the effect (e.g., footfall, topographic formation of the station, etc.). On the other hand, our intention was never to fully understand offenders’ decision-making processes and how people perceive deterrence cues generated by private security, or the interplay with these additional factors—which would serve as incredibly informative studies in the future. We were more interested in behavioural adaptations, which result from a clearly-defined intervention. The ‘why’ question remains unanswered; we argue that security guards caused both crime reductions and detection of offenders, but we cannot describe the micro-mediation of the causal mechanism that plays part here [117]. Therefore, while we interpret the evidence to suggest that the threat of sanctions is causally related to crime prvention, we would like to see more research on decision-making processes—as we would recommend for all hot spots experiments more generally. Such studies, for example, would directly debunk the displacement argument, or provide alternative explanations to such findings.

Another clear limitation is that our conclusion about the dosage outcome hypothesis is correlational, rather than causal. We show that as the number of patrols increase, crime appears to go down (see Figs 5 and 6). However, we did not randomly assign varying dosages, which leaves room for alternative hypotheses for these patterns and distributions. Our findings do not allow us to show firmly what the optimal number of security guards might be, for these are public spaces [116]. Nor do they inform us what the optimal length of time for each visit, or how many visits a security guard should make to each target location. There may be varying types of crime control strategies or bespoke engagement tactics for different locations [118, 119], but our study does not address. As Sherman (2013) argued for policing scholarship more broadly, the degree of sophistication of the available evidence has been quite limited; instead, we are blinded as to the ‘how much’ and the ‘what’ the police actually do out there. We urge crime control theorists and practitioners to consider these implications, as they carry wide consequences.

## Conclusion

Preventative private policing in public spaces is causally linked to both reductions in prevalence of victims as well as higher counts of offenders detected. Security guards, who are dynamic actors of social control mechanism, deter offenders. They prevent crime but also increase detection of new crimes. The classic role of security agents to ‘observe and report’ offers a limited view of security guards, as our findings indicate the potential of non-state agents to take an active part in the diversification of the modern criminal justice system portfolio.
